# Multiple Complications Following Acute Myocardial Infarction: A Case Report

**DOI:** 10.7759/cureus.104436

**Published:** 2026-02-28

**Authors:** Marco Antonio Rodríguez Sánchez, David Alejandro González Carrillo, Cristina Michelle Lugo Díaz, Omar Enrique Morales Flores, Luis David Beltrán Ontiveros, Jesus Miguel Figueroa Zaldivar, Pedro Antonio Nieto Morga

**Affiliations:** 1 Department of Internal Medicine, Center for Research and Teaching in Health Sciences, Autonomous University of Sinaloa, Civil Hospital of Culiacán, Culiacán, MEX; 2 Department of Cardiology, Center for Research and Teaching in Health Sciences, Autonomous University of Sinaloa, Civil Hospital of Culiacán, Culiacán, MEX

**Keywords:** acute myocardial infarction, cardiac tamponade, intracardiac thrombus, left ventricular aneurysm, stent thrombosis

## Abstract

Acute myocardial infarction (AMI) remains a leading cause of morbidity and mortality worldwide. Despite advances in early reperfusion and percutaneous coronary intervention (PCI), a subset of patients develops severe arrhythmic, thrombotic, and mechanical complications that markedly worsen prognosis and require rapid recognition and multidisciplinary management.

We describe a 66-year-old man with multiple cardiovascular risk factors who presented with an anteroseptal ST-elevation myocardial infarction and underwent urgent PCI with stent implantation in the left anterior descending artery. Eighteen hours later, he developed recurrent ischemia due to acute stent thrombosis, consistent with type 4b myocardial infarction, requiring repeat coronary intervention. His subsequent intensive care unit course was complicated by atrial fibrillation, acute ischemic heart failure, and progressive hemodynamic instability. Bedside transthoracic echocardiography identified a left ventricular aneurysm with intracavitary thrombus, severely reduced left ventricular ejection fraction, and a large pericardial effusion causing cardiac tamponade. Emergency surgical pericardial window confirmed hemopericardium. After prompt surgical intervention and optimization of guideline-directed medical therapy, the patient achieved clinical stabilization and was discharged with close follow-up.

This case highlights the dynamic and multifaceted nature of AMI, emphasizing that even with timely reperfusion, patients may develop a cascade of life-threatening complications. Early clinical vigilance, repeated imaging, and coordinated multidisciplinary care are essential to improve outcomes in complex post-infarction presentations.

## Introduction

Acute myocardial infarction (AMI) remains a leading cause of cardiovascular morbidity and mortality worldwide. Although early reperfusion strategies and advances in pharmacological therapy have significantly reduced short-term mortality, patients remain at risk for a spectrum of early post-infarction complications that may substantially worsen prognosis [1].

These complications can be broadly categorized as ischemic, electrical, and mechanical. Ischemic complications include recurrent myocardial infarction and acute stent thrombosis, a rare but catastrophic event typically occurring within the first 24 hours after percutaneous coronary intervention (PCI). Stent thrombosis results from a complex interplay of endothelial injury, high thrombotic burden, stent malapposition or underexpansion, and patient-related prothrombotic factors, leading to abrupt vessel occlusion and recurrent myocardial injury [2,3].

Electrical complications such as atrial fibrillation are common in the early post-AMI period and are associated with hemodynamic deterioration, heart failure, and increased thromboembolic risk. However, mechanical complications remain among the most life-threatening sequelae of AMI [1,4]. These include ventricular aneurysm formation, left ventricular thrombus, papillary muscle dysfunction, ventricular septal rupture, and free wall rupture. True ventricular aneurysm typically develops after extensive transmural infarction and is characterized by broad-based dyskinetic remodeling of infarcted myocardium, predisposing to heart failure and intracavitary thrombus formation [5,6].

Pericardial effusion and cardiac tamponade in the post-infarction setting warrant urgent evaluation for mechanical complications, particularly ventricular free wall rupture or pseudoaneurysm. Hemorrhagic pericardial effusion may result from contained myocardial rupture, microvascular hemorrhage, inflammatory pericardial involvement, or, less commonly, procedure-related vascular injury [7]. Rapid identification through multimodality imaging is essential, as these entities carry high mortality if not promptly recognized and treated.

While individual post-infarction complications are well described, the occurrence of multiple severe thrombotic, electrical, and mechanical complications within the same early post-AMI period is uncommon in the era of contemporary reperfusion therapy. This case illustrates a rare cascade of early post-infarction complications and underscores the importance of close hemodynamic monitoring, repeated imaging, and multidisciplinary management during the first 24 to 72 hours following AMI.

## Case presentation

**Figure 1:**
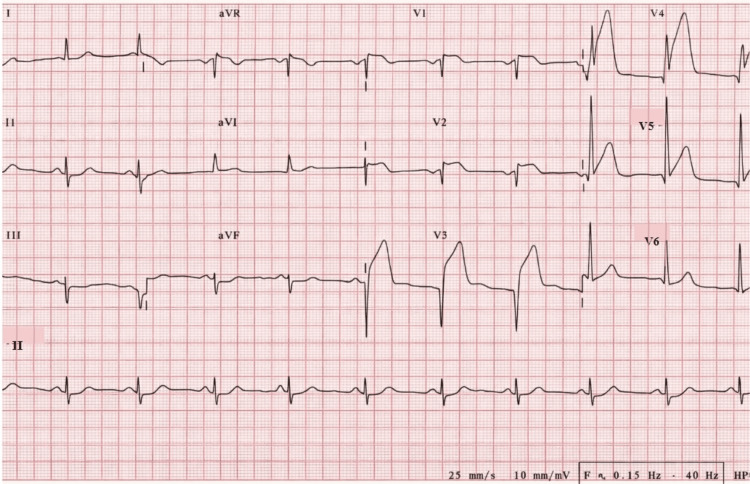
Initial 12-lead electrocardiogram demonstrating acute anteroseptal ST-segment elevation A 12-lead electrocardiogram obtained at initial presentation demonstrating acute ST-segment elevation in leads V1–V4, consistent with anteroseptal myocardial injury. No fully developed pathological Q waves are observed, supporting early transmural ischemia. These findings prompted urgent coronary angiography and primary percutaneous coronary intervention.

A 66-year-old man with multiple cardiovascular risk factors, including obesity, poorly controlled hypertension, heavy smoking, and chronic alcohol consumption, presented with acute, oppressive chest pain. Initially, the electrocardiogram showed acute anteroseptal ST-segment elevation in leads V1-V4 without pathological Q waves, consistent with early transmural injury (Figure 1). These findings prompted urgent coronary angiography with implantation of two stents in the left anterior descending (LAD) artery.

**Figure 2:**
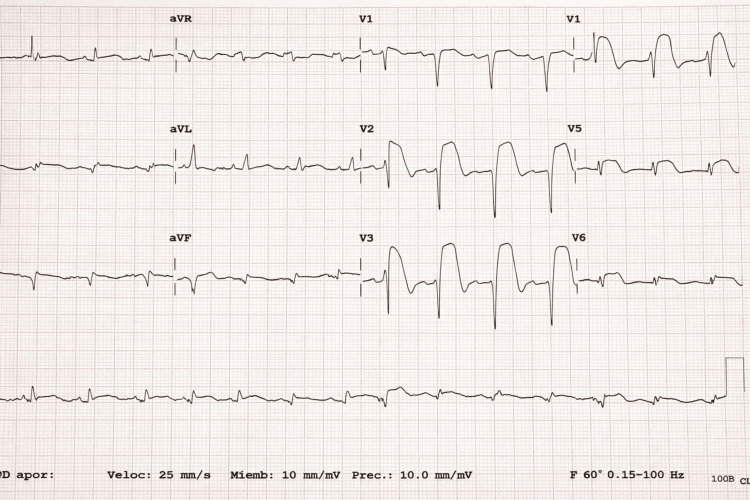
A 12-lead electrocardiogram showing recurrent anterior ST-segment elevation Electrocardiogram demonstrating extensive ST-segment elevation in the anterior leads, consistent with acute anterior myocardial injury. In the clinical context of recent percutaneous coronary intervention, these findings were highly suggestive of acute stent thrombosis (type 4b myocardial infarction).

Eighteen hours after the initial PCI, the patient experienced recurrent oppressive chest pain associated with diaphoresis and palpitations. The electrocardiogram showed recurrent anterior ST-segment elevation with associated Q waves (Figure 2), and cardiac biomarkers were markedly elevated (five times the upper reference limit of the 99th percentile). A transthoracic echocardiogram performed in the first few hours after admission showed regional wall motion abnormalities in the left ventricular (LAD) artery territory with moderately reduced left ventricular systolic function (LVEF of approximately 45%), without pericardial effusion or intracavitary thrombus at that time. Urgent repeat coronary angiography confirmed acute stent thrombosis (type 4b myocardial infarction).

**Figure 3:**
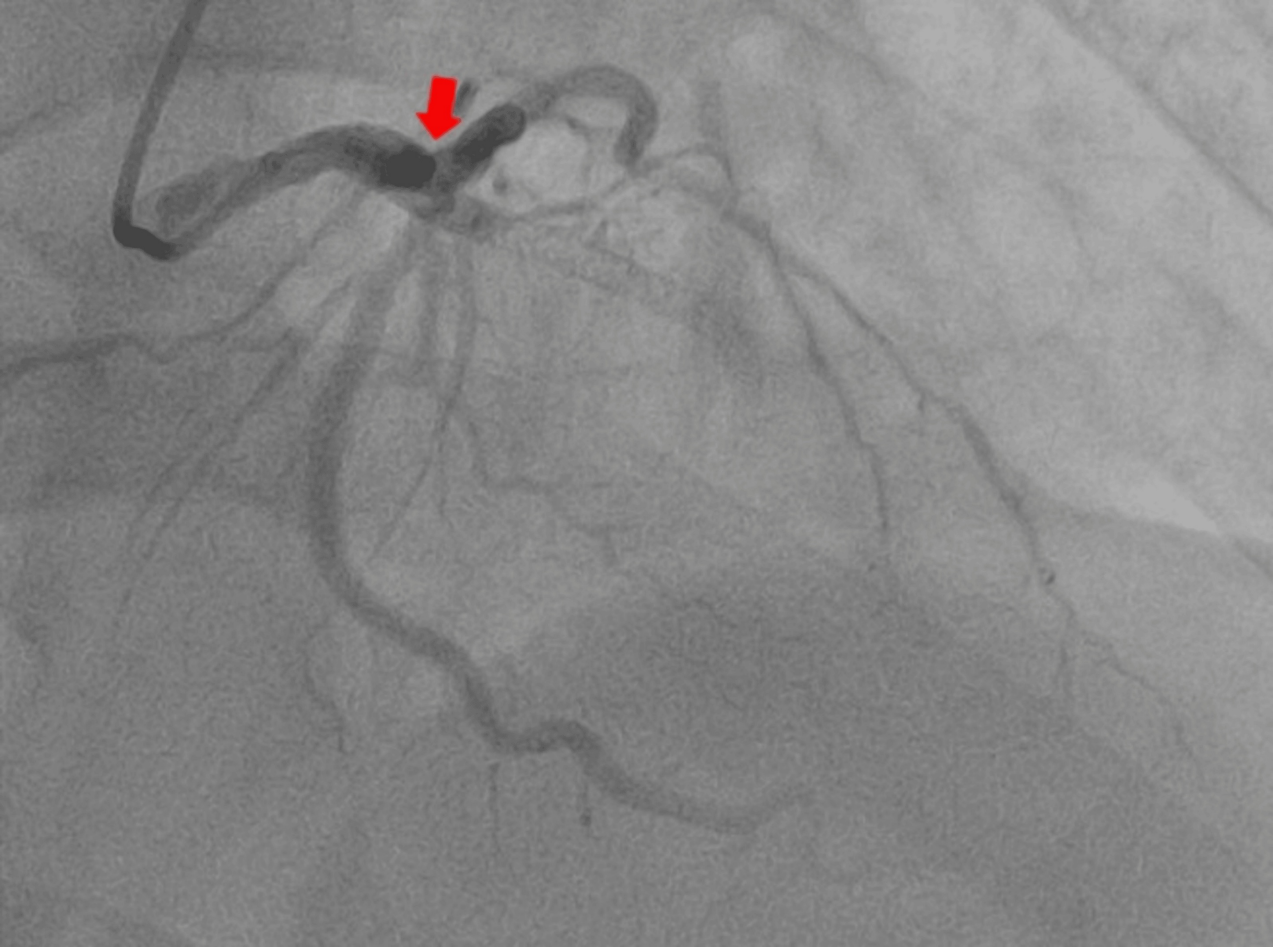
Selective coronary angiography demonstrating acute LAD stent thrombosis and critical distal circumflex stenosis Coronary angiography demonstrating complete thrombotic occlusion proximal to the previously implanted stent in the LAD artery (red arrow), along with a critical 90% stenosis in the distal segment of the left circumflex artery. LAD: Left anterior descending.

Coronary angiography revealed complete thrombotic occlusion proximal to the previously implanted LAD stent and critical stenosis of 90% of the distal circumflex artery (Figure 3).

Repeat PCI was successfully performed with implantation of two drug-eluting stents in the LAD and one in the distal circumflex artery, achieving optimal angiographic flow restoration. During repeat PCI, the patient received an intracoronary bolus of tirofiban followed by a 12-hour intravenous infusion. Intravenous unfractionated heparin was administered during the procedure. Acute stent thrombosis was considered likely multifactorial, potentially related to high thrombotic burden, endothelial injury, and mechanical factors such as stent underexpansion or vessel tortuosity.

Following stabilization, the patient was admitted to the intensive care unit (ICU) for close hemodynamic monitoring. On admission, he was tachycardic (>130 bpm), and electrocardiography demonstrated atrial fibrillation without hemodynamic compromise. Anticoagulation was transitioned to therapeutic-dose enoxaparin due to the coexistence of left ventricular thrombus and atrial fibrillation. Dual antiplatelet therapy (aspirin plus clopidogrel) and high-intensity statin therapy were continued throughout hospitalization.

**Figure 4:**
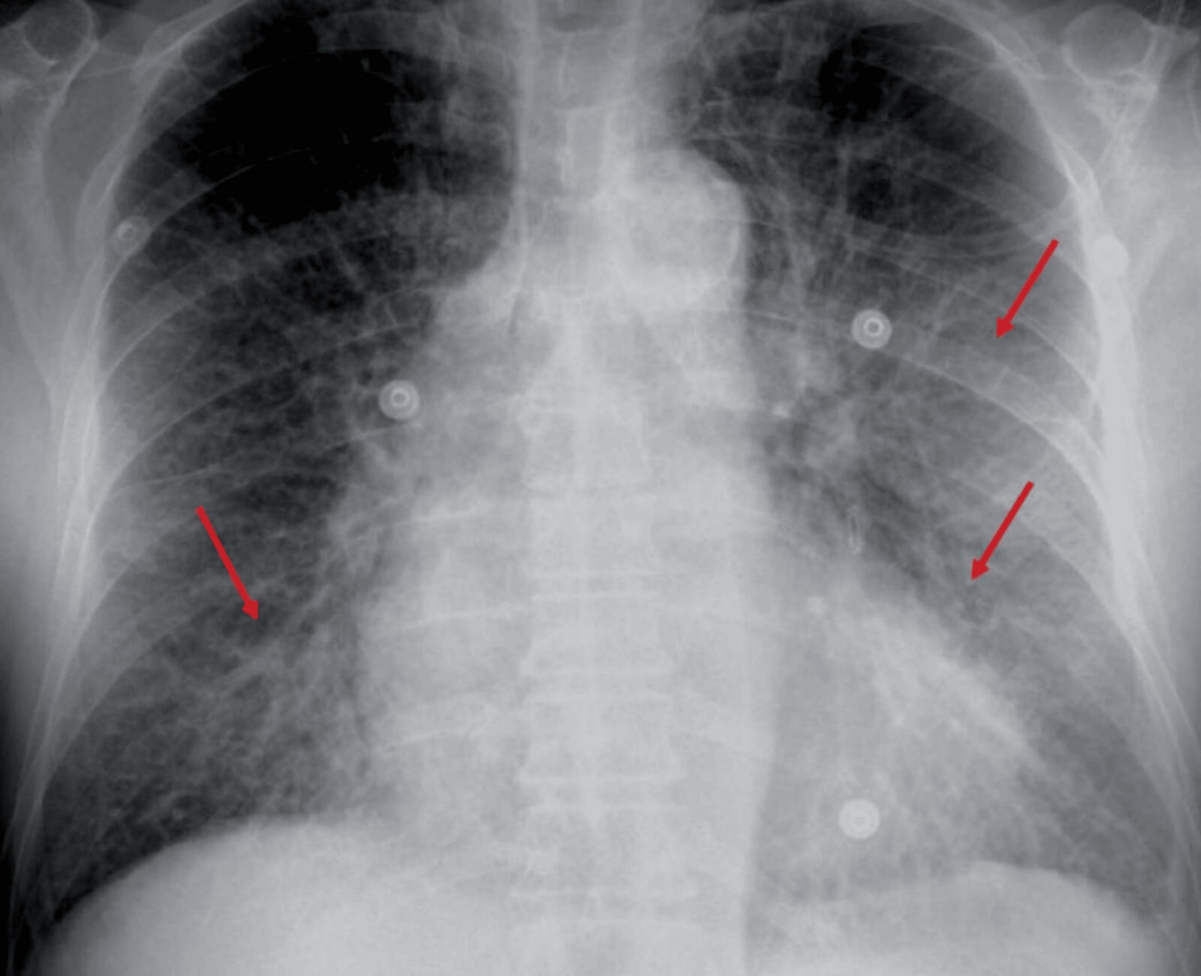
Chest radiograph demonstrating pulmonary congestion and bilateral pleural effusions Chest radiograph demonstrating pulmonary vascular congestion (red arrows) and bilateral pleural effusions. In the clinical context of peripheral edema and markedly elevated NT-proBNP levels, these findings support the diagnosis of acute ischemic heart failure.

During hospitalization in the ICU, the patient developed progressive dyspnea and orthopnea. Chest radiography showed pulmonary congestion with bilateral pleural effusions (Figure 4). Along with peripheral edema and markedly elevated NT-proBNP levels (9,999 pg/mL), these findings were consistent with acute ischemic heart failure secondary to extensive myocardial injury and adverse ventricular remodeling. Intravenous loop diuretics were administered for five days, resulting in significant clinical improvement, with resolution of pulmonary congestion and peripheral edema. Oral diuretics were subsequently continued until hospital discharge, in accordance with current clinical guidelines.

Within the first 12 hours of ICU admission, the patient developed progressive hypotension (blood pressure decreased to 85/50mmHg), persistent tachycardia (>130 bpm), and muffled heart sounds. Oxygen saturation remained 90%, and urine output declined. These findings raised concern for mechanical complications and prompted urgent bedside transthoracic echocardiography.

**Figure 5:**
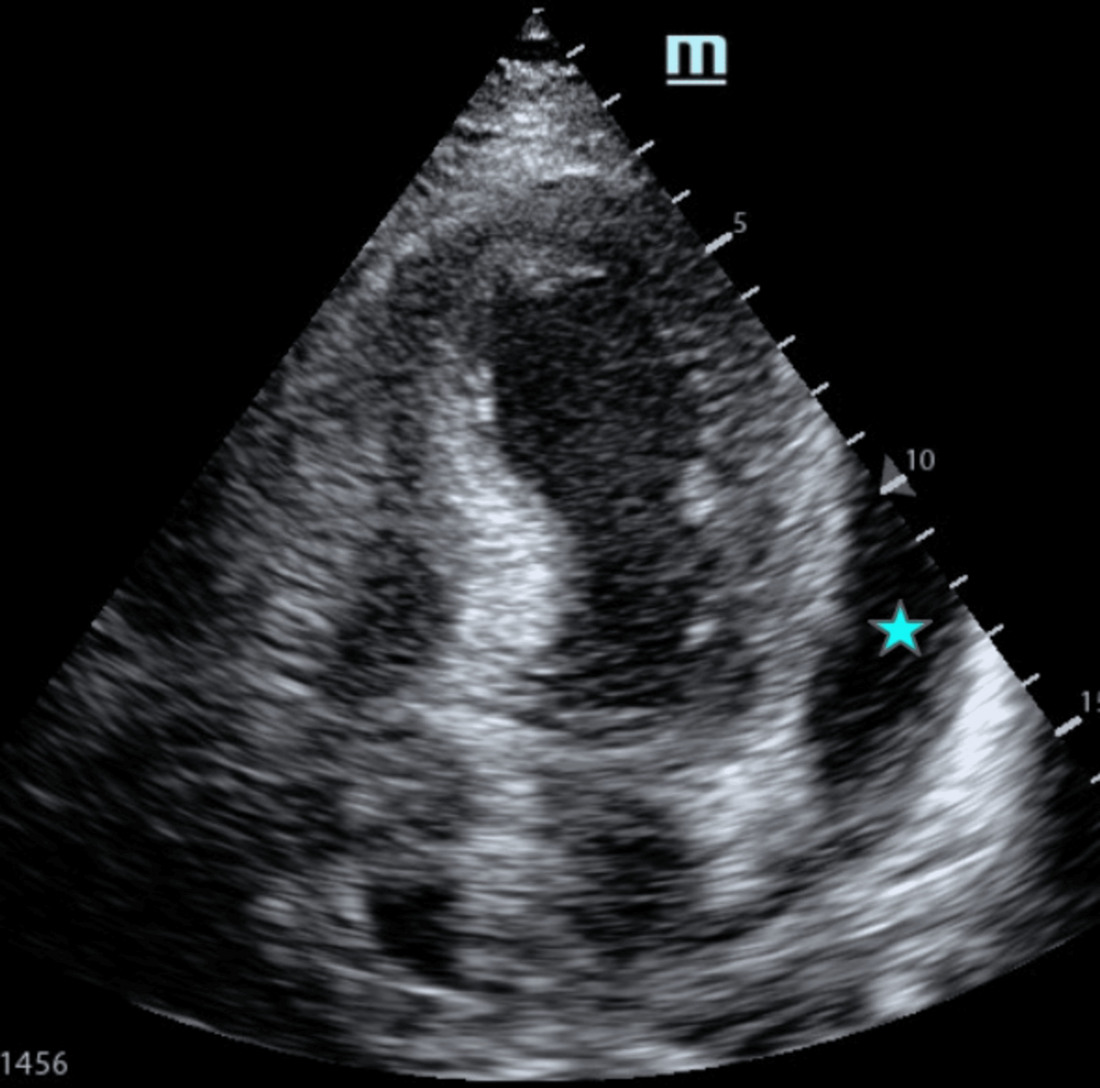
Apical four-chamber view demonstrating a left ventricular aneurysm and an intracavitary thrombus Transthoracic echocardiography in the apical four-chamber view demonstrating apical aneurysmal dilation of the left ventricle with an associated intracavitary thrombus. A circumferential pericardial effusion is also noted (blue asterisk).

**Figure 6:**
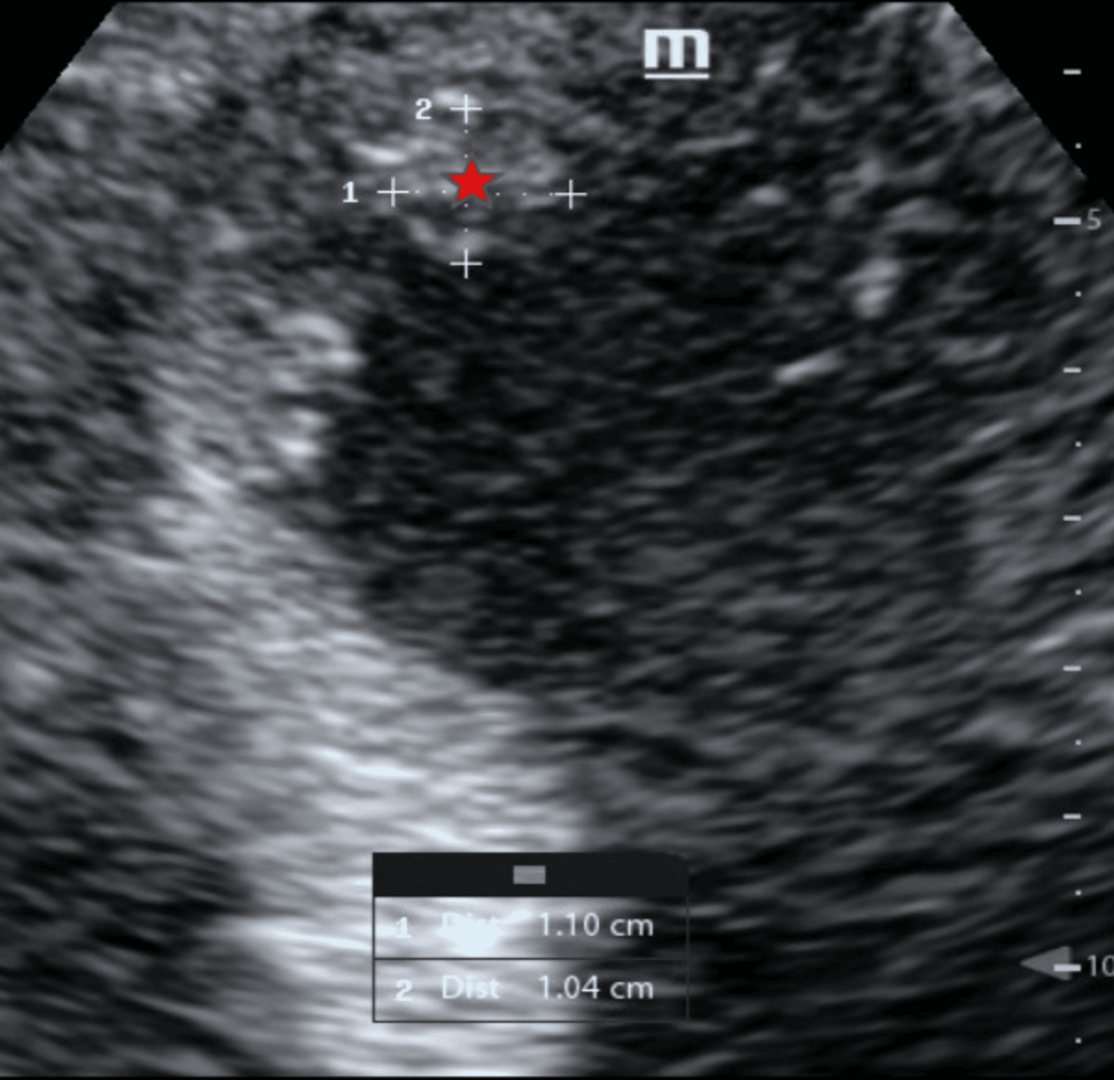
Apical four-chamber view (focused apical imaging) demonstrating a left ventricular intracavitary thrombus Transthoracic echocardiography with focused imaging of the left ventricular apex demonstrating an intracavitary thrombus attached to the endocardial wall, measuring approximately 1.10 × 1.04 cm (red asterisk).

**Figure 7:**
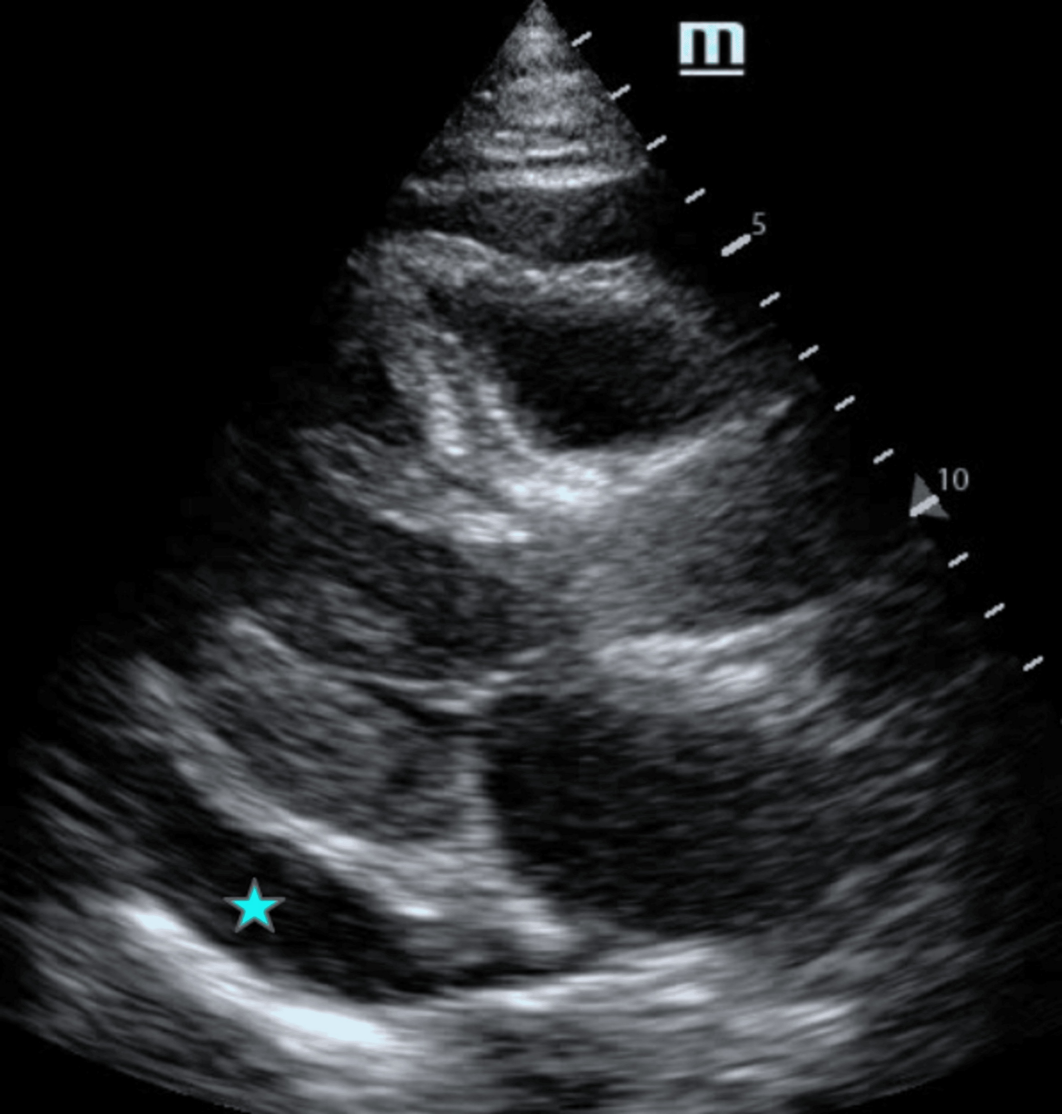
Parasternal long-axis view demonstrating a circumferential pericardial effusion Transthoracic echocardiography in the parasternal long-axis view demonstrating a circumferential pericardial effusion (blue asterisk) with evidence of right-sided chamber compression.

**Figure 8:**
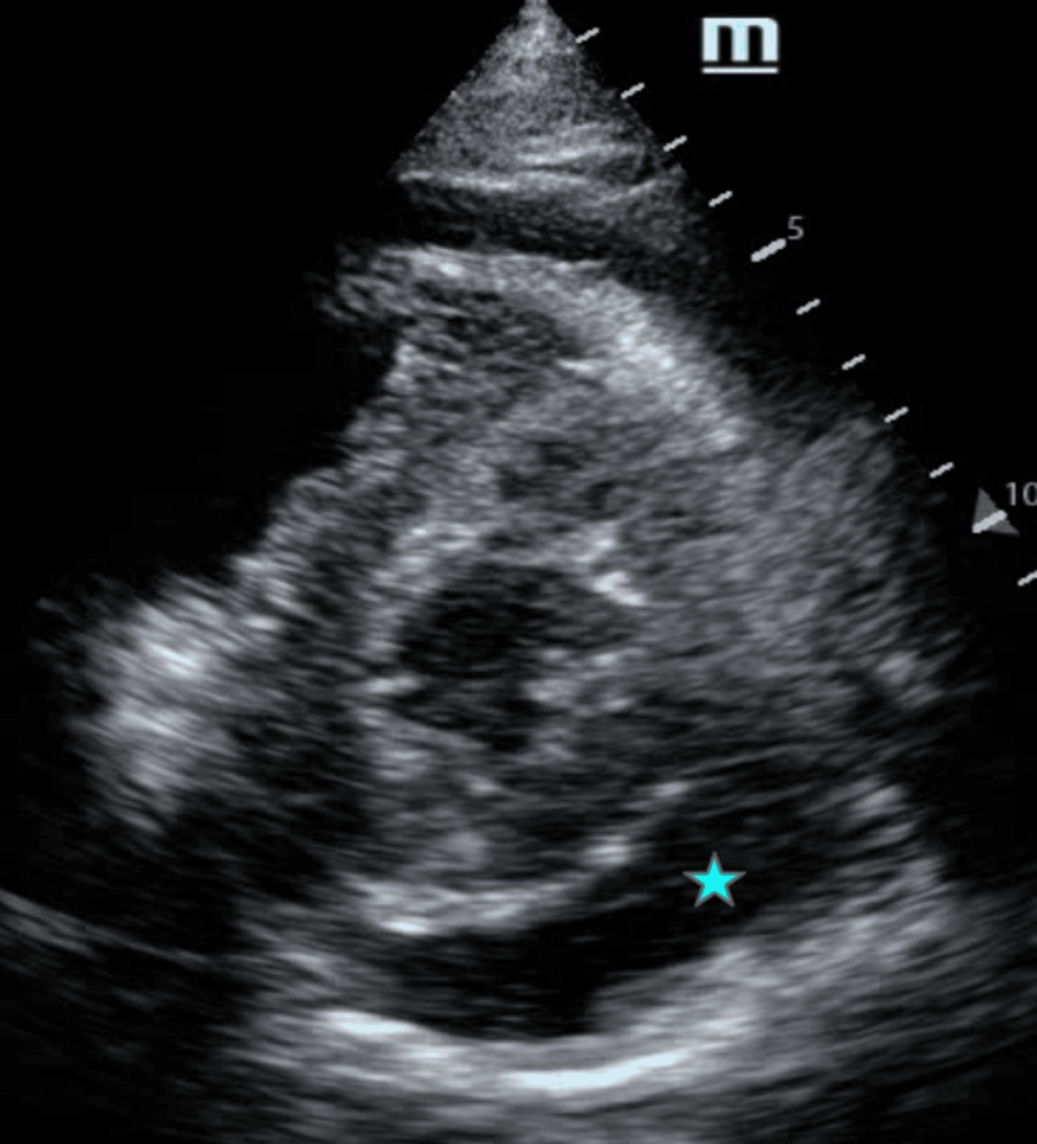
Parasternal short-axis view demonstrating a circumferential pericardial effusion (maximum diameter, 2 cm) Transthoracic echocardiography in the parasternal short-axis view demonstrating a circumferential pericardial effusion (blue asterisk) with an estimated maximum separation of approximately 2 cm between the pericardial layers.

Echocardiographic evaluation (Figures 5-8) revealed a left ventricular aneurysm with an intracavitary thrombus, a reduced left ventricular ejection fraction of 37%, and regional wall motion abnormalities in the LAD territory. A circumferential pericardial effusion measuring approximately 2 cm was identified, with features of cardiac tamponade physiology, including right atrial and right ventricular diastolic collapse and a 30% respiratory variation in transmitral inflow.

Given the presence of hemopericardium in the post-infarction setting, the differential diagnosis included ventricular free wall rupture with contained pseudoaneurysm formation versus hemorrhagic effusion related to anticoagulation or post-infarction inflammatory processes. However, imaging findings demonstrated preserved myocardial wall continuity and aneurysmal remodeling rather than a narrow-necked contained rupture, favoring a post-infarction ventricular aneurysm complicated by hemopericardium.

An urgent cardiothoracic surgery consultation was obtained, and emergent pericardial window surgery was performed. Intraoperatively, 500 mL of hemorrhagic pericardial fluid consistent with hemopericardium was drained and sent for cytological, biochemical, and microbiological analysis. A mediastinal drain was placed without complications.

Postoperatively, the patient remained hemodynamically stable with progressive clinical improvement. Antithrombotic therapy was carefully reassessed in light of the recent PCI, left ventricular thrombus, atrial fibrillation, and hemorrhagic pericardial effusion. Guideline-directed medical therapy for heart failure with reduced ejection fraction was optimized, including sacubitril/valsartan, dapagliflozin, and spironolactone, all of which were well tolerated.

After a progressive reduction in mediastinal drainage output, the drain was removed. The patient was transferred from the ICU to the cardiothoracic surgery service and discharged home after two additional days of stable clinical evolution.

At discharge, the patient was scheduled for structured outpatient follow-up with the cardiology and cardiothoracic surgery departments. Surveillance includes clinical reassessment at two weeks, repeat transthoracic echocardiography at three months to evaluate left ventricular remodeling and thrombus resolution, rhythm monitoring if symptomatic, and reassessment of anticoagulation strategy based on imaging findings. The patient was counseled regarding warning symptoms requiring urgent evaluation. Although short-term stabilization was achieved, long-term prognosis remains guarded given the extent of myocardial injury, and continued surveillance is warranted.

## Discussion

This case illustrates a complex and interrelated cascade of ischemic, mechanical, thrombotic, and electrical complications occurring in the early phase following extensive ST-segment elevation myocardial infarction (STEMI). Rather than representing isolated events, acute stent thrombosis, left ventricular (LV) aneurysm with thrombus formation, acute heart failure, and hemopericardium with tamponade physiology developed within a unified pathophysiological framework driven by large transmural myocardial injury.

Acute stent thrombosis (type 4b myocardial infarction) is a rare but catastrophic complication that most frequently occurs within the first 24 hours after PCI and is associated with recurrent ischemia, hemodynamic instability, and high mortality if not promptly treated [2]. In this patient, the mechanism was likely multifactorial. Contributing factors may have included a high thrombotic burden, endothelial disruption, mechanical considerations such as stent underexpansion or vessel geometry, and the pro-inflammatory, prothrombotic milieu associated with extensive myocardial necrosis. Even in the absence of antiplatelet therapy interruption, the systemic hypercoagulable state following a large infarction may predispose to acute thrombotic events.

The subsequent development of an LV aneurysm with intracavitary thrombus reflects adverse ventricular remodeling following transmural anterior infarction. Infarct expansion, wall thinning, and regional akinesia promote blood stasis and fulfill Virchow’s triad, increasing thrombus formation risk [3]. Although the incidence of LV thrombus has declined in the era of early reperfusion, it remains clinically relevant in patients with large infarcts and reduced left ventricular ejection fraction [4]. The presence of LV thrombus significantly increases the risk of systemic embolization, including cerebrovascular and peripheral arterial events [5]. Transthoracic echocardiography remains the first-line diagnostic modality, with cardiac magnetic resonance imaging offering greater sensitivity in selected cases [4]. Anticoagulation is typically recommended for at least three months, with duration guided by repeat imaging and individualized thromboembolic and bleeding risk assessment.

Acute heart failure in this context reflects the combined effects of extensive myocardial loss, neurohormonal activation, and progressive ventricular remodeling [6]. Early initiation and optimization of guideline-directed medical therapy for heart failure with reduced ejection fraction are essential to mitigate adverse remodeling and improve long-term outcomes [8].

One of the most critical aspects of this case was the rapid progression to cardiac tamponade due to hemopericardium. Transthoracic echocardiography confirmed a large pericardial effusion with right heart collapse (transthoracic echocardiographic findings are summarized in Table 1). In the post-infarction setting, hemorrhagic pericardial effusion can result from various mechanisms, including microvascular myocardial hemorrhage, inflammatory involvement of the pericardium, bleeding associated with anticoagulation, or, less frequently, rupture of the ventricular free wall [9]. The presence of hemopericardium necessitates careful distinction between a true ventricular aneurysm and a pseudoaneurysm, the latter representing a contained myocardial rupture with a substantially higher risk of catastrophic rupture and mortality.

**Table 1:** Summary of transthoracic echocardiographic findings

Parameters	Findings	Details
Left ventricular morphology	Left ventricular aneurysm	Associated with intracavitary thrombus
Left ventricular systolic function	Reduced	Left ventricular ejection fraction: 37%
Regional wall motion	Abnormal	Hypokinesia in the left anterior descending (LAD) artery territory
Pericardial effusion	Present	Global pericardial effusion measuring 2 cm
Hemodynamic impact	Cardiac tamponade physiology	Right chamber collapse and 30% respiratory variation in transmitral flow

Cardiac pseudoaneurysm remains an essential differential diagnosis in post-infarction patients with pericardial effusion. As reported by Lorusso et al., pseudoaneurysms are frequently located in the inferior or posterolateral wall and carry a guarded prognosis, with survival rates near 40% and significant mortality related to arrhythmias or heart failure [10]. In the present case, multimodality imaging demonstrated preserved myocardial wall continuity and broad-based aneurysmal remodeling rather than a narrow-necked contained rupture, supporting the diagnosis of post-infarction ventricular aneurysm rather than pseudoaneurysm. Nevertheless, its consideration was essential in guiding urgent surgical evaluation.

Importantly, cardiogenic shock was strongly considered in the differential diagnosis of the patient's hemodynamic deterioration. However, bedside echocardiography demonstrated preserved ventricular wall continuity and identified tamponade physiology as the primary reversible cause of instability. The rapid hemodynamic improvement following pericardial drainage further supported obstructive shock secondary to cardiac tamponade rather than primary pump failure.

An additional therapeutic complexity in this case involved balancing ischemic and bleeding risks in a patient requiring dual antiplatelet therapy following recent PCI, anticoagulation for LV thrombus and atrial fibrillation, and management of hemorrhagic pericardial effusion. This highlights the necessity of individualized antithrombotic strategies and dynamic reassessment as clinical conditions evolve.

The favorable outcome in this patient underscores the importance of early recognition of overlapping post-infarction complications, repeated multimodal imaging, and coordinated multidisciplinary management involving interventional cardiology, critical care, and cardiothoracic surgery.

In summary, this case emphasizes that early post-STEMI complications may occur as an interconnected cascade rather than isolated events. Acute stent thrombosis, ventricular remodeling with thrombus formation, heart failure progression, and hemopericardium require rapid identification and integrated management strategies. Careful diagnostic differentiation between ventricular aneurysm and pseudoaneurysm is crucial, as it significantly influences prognosis and therapeutic decision-making. Continued research is needed to refine anticipatory strategies and optimize individualized care in complex post-infarction patients.

## Conclusions

This case highlights the complex and potentially life-threatening spectrum of post-AMI complications, including ischemic (acute stent thrombosis), mechanical (ventricular remodeling, aneurysm formation, and hemopericardium), and electrical instability, even after timely reperfusion therapy. The clinical course underscores how these complications may coexist and interact within a unified pathophysiological cascade following extensive myocardial injury.

Importantly, the presence of hemorrhagic pericardial effusion in the post-infarction setting necessitates careful differentiation between a true ventricular aneurysm and a cardiac pseudoaneurysm, as the latter carries a significantly higher risk of rupture and mortality. In our case, a pseudoaneurysm was thoroughly considered in the differential diagnosis; however, multimodality imaging findings supported a post-infarction ventricular aneurysm with secondary complications rather than a contained myocardial rupture. This case emphasizes the critical role of close hemodynamic surveillance, repeated multimodal imaging, and coordinated multidisciplinary management in patients with complicated myocardial infarction. Early recognition of overlapping ischemic, mechanical, and thrombotic processes is essential to guide individualized therapeutic strategies and improve outcomes.
